# 2-Deoxy-d-Glucose Can Complement Doxorubicin and Sorafenib to Suppress the Growth of Papillary Thyroid Carcinoma Cells

**DOI:** 10.1371/journal.pone.0130959

**Published:** 2015-07-02

**Authors:** Shuo-Yu Wang, Yau-Huei Wei, Dar-Bin Shieh, Li-Ling Lin, Shih-Ping Cheng, Pei-Wen Wang, Jiin-Haur Chuang

**Affiliations:** 1 Department of Pediatrics, Chi-Mei Medical Center, Tainan, Taiwan; 2 Graduate Institute of Clinical Medical Sciences, Chang-Gung University, College of Medicine, Kaohsiung, Taiwan; 3 Department of Biochemistry and Molecular Biology, National Yang-Ming University, Taipei, Taiwan; 4 Department of Medicine, Mackay Medical College, New Taipei City, Taiwan; 5 Institute of Basic Medical Sciences, National Cheng Kung University, Tainan, Taiwan; 6 Department of Medical Research, Kaohsiung Chang Gung Memorial Hospital, Kaohsiung, Taiwan; 7 Department of Surgery, Division of Pediatric Surgery, Kaohsiung Chang Gung Memorial Hospital, Kaohsiung, Taiwan; 8 Department of Surgery, Mackay Memorial Hospital, Taipei, Taiwan; 9 Department of Internal and Nuclear Medicine, Kaohsiung Chang Gung Memorial Hospital, Kaohsiung, Taiwan; Universite Libre de Bruxelles (ULB), BELGIUM

## Abstract

Tumor cells display a shift in energy metabolism from oxidative phosphorylation to aerobic glycolysis. A subset of papillary thyroid carcinoma (PTC) is refractory to surgery and radioactive iodine ablation. Doxorubicin and sorafenib are the drugs of choice for treating advanced thyroid cancer but both induce adverse effects. In this study, we assessed the anti-cancer activity of 2-deoxy-d-glucose (2-DG) alone and in combination with doxorubicin or sorafenib in PTC cell lines with (BCPAP) and without (CG3) the *BRAF^V600E^* mutation. BCPAP cells were more glycolytic than CG3 cells, as evidenced by their higher extracellular l-lactate production, lower intracellular ATP level, lower oxygen consumption rate (OCR), and lower ratio of OCR/extracellular acidification rate. However, dose-dependent reduction in cell viability, intracellular ATP depletion, and extracellular l-lactate production were observed after 2-DG treatment. Regression analysis showed that cell growth in both cell lines was dependent on ATP generation. 2-DG increased the chemosensitivity of BCPAP and CG3 cell lines to doxorubicin and sorafenib. These results demonstrate that the therapeutic effects of low combined doses of 2-DG and doxorubicin or sorafenib are similar to those of high doses of doxorubicin or sorafenib alone in PTC cell lines regardless of the *BRAF^V600E^* mutation.

## Introduction

One of the fundamental biochemical differences between malignant tumor and non-tumor cells is a shift in energy metabolism from oxidative phosphorylation (OXPHOS) to aerobic glycolysis, also known as the Warburg effect [1–3]. Even in the presence of oxygen, tumor cells predominantly use glycolysis, with reduced mitochondrial OXPHOS, for the synthesis of ATP, and exhibit increased glucose consumption that is facilitated by glucose transporters [4,5]. Therefore, new therapeutic approaches have recently emerged that target multiple bioenergetic pathways combined with conventional, “standard-of-care” chemotherapeutics in tumor cells [6–10].

Papillary thyroid carcinoma (PTC) is the most common form of well-differentiated thyroid cancer [11]. Although PTC tends to have a favorable prognosis overall, a subset of these tumors is refractory to surgery and to radioactive iodine ablation [12]. Patients with advanced PTC have been treated with external beam radiation and chemotherapy. Before November 2013, doxorubicin, a cytotoxic drug, was the only systemic agent approved by the United States Food and Drug Administration (US FDA) for the treatment of thyroid cancer [13]. However, previous studies [14–16] have reported only modest response rates and short durations of therapeutic benefit from doxorubicin, and that its dose-dependent cardiotoxicity culminates in congestive heart failure, which has clearly limited its use. In November 2013, the US FDA approved the use of sorafenib, an oral multi-kinase inhibitor for the treatment of differentiated thyroid cancer metastases unresponsive to radioiodine therapy [17]. Sorafenib targets B-type Raf kinase (BRAF), including both wild-type and *BRAF* ^*V600E*^ (the major mutation of PTC), as well as VEGFR1, VEGFR2, VEGFR3, PDGFRβ, and RET (also RET/PTC) [18]. In a phase III clinical trial, it significantly improved progression-free survival compared to placebo in patients with progressive radioactive iodine-refractory differentiated thyroid cancer, but adverse events were consistent with the known safety profile of sorafenib [18].

The metabolic inhibitor 2-deoxy-d-glucose (2-DG) is a synthetic glucose analog whose antitumor activity has been demonstrated in various cancer cell lines and in *in vivo* murine cancer models [19–25]. 2-DG also increases the antitumor activity of doxorubicin in cell culture [25] and in tumor-bearing mice [22]. In addition, 2-DG is one of the first compounds known to mimic the beneficial effects of caloric restriction [26,27]. It prevents neurodegeneration in cell culture [28] and in the brain of animals subjected to a variety of insults, including an inducer of Parkinsonism [29]. Positive effects of 2-DG have also been reported in a transgenic model of Alzheimer’s disease [30] and for the treatment of electrically induced epileptic seizure [31]. Moreover, 2-DG has been reported to be safe and its antitumor effects have been demonstrated in a phase I/II clinical trial involving patients with recurrent solid tumors [32,33]. The most common adverse events from 2-DG administration are fatigue, sweating, dizziness, and nausea, thus mimicking hypoglycemic symptoms. The most serious adverse effects at more than 60 mg/kg doses are hyperglycemia, gastrointestinal bleeding and grade 3 corrected QT interval prolongation, which can be reversed by cessation of 2-DG treatment [32,33]. Based on the above evidence, we hypothesized that 2-DG combined with doxorubicin or sorafenib could inhibit the growth of two PTC cell lines: BCPAP (expressing the *BRAF* ^*V600E*^ mutation) and CG3 (lacking the *BRAF* ^*V600E*^ mutation).

## Materials and Methods

### Cell lines and cell culture

The PTC cell line CG3 was kindly provided by Dr. Jen-Der Lin (Chang Gung Memorial Hospital, Taipei, Taiwan) and was cultured as previously described [34]. BCPAP cell lines were purchased from the German Collection of Microorganisms and Cell Cultures (Leibniz-Institut DSMZ-Deutsche Sammlung von Mikroorganismen und Zellkulturen GmbH, Braunschweig, Germany). The cells were cultured in complete RPMI-1640 medium supplemented with 10% fetal bovine serum (FBS), streptomycin (104 μg/mL), penicillin (104 U/mL), amphotericin B (250 μg/mL) (Tebu-bio, Le-Perray-en-Yvelines, France) and L-glutamine (20 mM) (Invitrogen, Carlsbad, CA, USA). All cells were grown at 37°C in a humidified 5% CO_2_ atmosphere.

### Chemicals

2-DG, doxorubicin, and sorafenib were purchased from Sigma-Aldrich Chemical Co. (St. Louis, MO, USA). 2-DG and doxorubicin were dissolved in water as 1 M and 10 mM stock, respectively. Sorafenib was dissolved in dimethylsulfoxide (DMSO) as 10 mM stock.

### Cell viability assay

Cell viability was assayed by using WST-1 kit (Roche Applied Science, Mannheim, Germany). Briefly, 5 × 10^3^ cells were plated in each well of a 96-well plate and cultured overnight. To evaluate the effect of 2-DG on cell viability, PTC cells were treated with different doses of 2-DG (0–4 mM), or with 0.0625 mM or 0.25 mM 2-DG plus doxorubicin (0–2 μM) or sorafenib (0–10 μM), after which the cells were incubated for another 48 h. The absorbance of the samples at 450 nm was measured using a 96-well plate reader (Tecan, Männedorf, Switzerland). All experiments were performed three times independently, each time in triplicate to confirm the results.

### Colony formation assay

Cells were trypsinized and plated in 6-well dishes at different densities depending on the potency of the treatments (from 5 × 10^3^ cells/well). The cells were allowed to attach overnight and then were treated with drugs at the corresponding dilution. Forty-eight hours after treatment, the medium was replaced with fresh medium, and the plates were incubated at 37°C. Three to five days later, the cells were fixed and stained with 10% methylene blue in 70% ethanol. The number of colonies was calculated (AlphaView SA 3.4.0; ProteinSimple, San Jose, CA, USA). Triplicate wells were set up for experiments done under each condition.

### Glucose uptake analysis

Cells (3 × 10^4^ cells) were seeded in a 96-well black and clear-bottomed plate with 100 μl of culture medium. After the cells had been incubated at 37°C and 5% CO_2_ overnight, the medium was replaced with glucose-free medium containing 150 μg/ml of 2-NBDG (a fluorescence-labeled deoxyglucose analog) (Cayman Chemical, Ann Arbor, MI, USA) The cells were exposed to 2-DG, doxorubicin, or sorafenib and incubated for 3 more h. The plate was centrifuged for 5 min at 400 × *g* at room temperature, the supernatant was aspirated, and then 200 μl of cell-based assay buffer was added and the plate was centrifuged again for 5 min at 400 × *g*. The supernatant was then aspirated and an additional 100 μl of cell-based assay buffer was added. The fluorescence at a wavelength of excitation/emission of 485/535 nm was measured using a plate reader (Thermo Scientific, Waltham, MA, USA).

### Intracellular ATP content analysis

The intracellular ATP content in the cells was determined using a luciferin-luciferase bioluminescence assay. Cells were plated in sterile white 96-well microplates (PerkinElmer, Waltham, MA, USA) at a density of 5 × 10^3^ cells per well. After they had been incubated overnight, the cells were treated with 2-DG or doxorubicin or both for 48 h and then lysed and processed (ATPlite Luminescence Assay system; PerkinElmer). Luminescent signals from each well were measured (Luminoskan Ascent Microplate Luminometer; Thermo Scientific). The results were normalized to the total protein level in the cell lysate. Three replicates were used for each group, and the experiments were repeated three times to confirm the results.

### Glycolysis assay

Extracellular l-lactate was quantified using a kit (Glycolysis Cell-Based Assay; Cayman Chemical, Ann Arbor, MI, USA). Briefly, cells were seeded into 96-well plates at a density of 5 × 10^3^ cells per well in 120 μL of medium and then cultured overnight in a CO_2_ incubator at 37°C. The cells were then incubated with 2-DG, doxorubicin, or both for 48 h, and10 μL of supernatant from cultured cell plates was then mixed with the kit reactants in a 96-well plate. Absorbance values at 490 nm were obtained using a plate reader (Tecan, Männedorf, Switzerland). Readings were corrected for background absorbance using the absorbance value from the culture medium only, and the corrected values were applied to a standard curve to calculate extracellular lactate levels. Three replicates were used for each group, and the experiments were repeated three times to confirm the results.

### Extracellular flux assay

A bioenergetic function assay [35] and a flux analyzer (XF96 Extracellular Flux Analyzer; Seahorse Bioscience, North Billerica, MA, USA) were used to determine the mitochondrial and glycolytic function in PTC cells. After they had been seeded and incubated overnight, the cells were washed with an unbuffered assay medium and treated with 2-DG (0–4 mM) for 3 h before the functions were measured. Both the oxygen consumption rate (OCR) and the extracellular acidification rate (ECAR) were measured over 1 h after the cells had been conditioned in the assay medium. Three replicates were used for each group, and the experiments were repeated three times to confirm the results.

### Flow cytometric analysis of early apoptosis

Briefly, the cells were treated with 2-DG, doxorubicin, sorafenib, or 2-DG plus doxorubicin or sorafenib for 48 h. They (1 × 10^6^) were then harvested (using trypsinization and centrifugation) and suspended in binding buffer. Apoptosis was assessed using the annexin V-FITC/propidium iodide (PI) double staining. An aliquot of 300 μl was incubated with 3 μl of annexin V-FITC or 3 μl of PI for 20 min at room temperature in the dark. The stained cells were analyzed using fluorescence-activated cell sorting (FACS) on a flow cytometer (FACSCanto II; BD Biosciences, Franklin Lakes, NJ, USA). The experiments were repeated three times to confirm the results.

### Western blot analysis

PTC cells were incubated with 2-DG, doxorubicin, or both for 48 h, and were lysed, and the lysates were cleared using centrifugation at 13,000 × g for 15 min at 4°C. After the cells had been lysed, the protein content was quantified using the Bradford's method. Equal amounts of protein were loaded to each lane for sodium dodecyl sulfate polyacrylamide gel electrophoresis (SDS-PAGE). The protein was transferred to a piece of polyvinylidene fluoride (PVDF) membrane (Immobilon-P; Millipore, Billerica, MA, USA) and incubated with 10% nonfat milk for 1 h. Membranes were then incubated with mouse anti-β*-*actin monoclonal antibody (mAb) (Millipore), rabbit anti-hexokinase II mAb (HKII), rabbit anti-pyruvate kinase isozyme M2 mAb (PKM2), rabbit anti-lactate dehydrogenase A mAb (LDH-A), and rabbit anti-caspase 3 mAb (Cell Signaling Technology, Inc., Danvers, MA, USA) overnight at 4°C, and then washed three times. After the cells had been washed, the membranes were incubated with diluted (1:10^3^) HRP-conjugated anti-mouse or anti-rabbit immunoglobulin G (IgG) antibody for 2 h at room temperature, washed three times for 5 min, and visualized using a horseradish peroxidase (HRP)-chemiluminescence detection kit and an imager (LAS-3000; Fujifilm, Tokyo, Japan). To quantify the protein signal intensity, the Image J software was used.

### Statistical analysis

All data presented in the figures represent the mean values obtained from at least three experiments. Data expressed as the mean ± standard deviation (SD) were used for comparative analysis between groups. Unless otherwise specified, PRISM 5.0 (GraphPad Software, San Diego, CA, USA), the half-maximal inhibitory concentration (IC_50_), Student's *t* test, two-way analysis of variance (ANOVA), and linear regression were used for data analysis. The difference is considered significance when *p* < 0.05.

## Results

### Mapping bioenergetics in PTC cells

**Fig 1:**
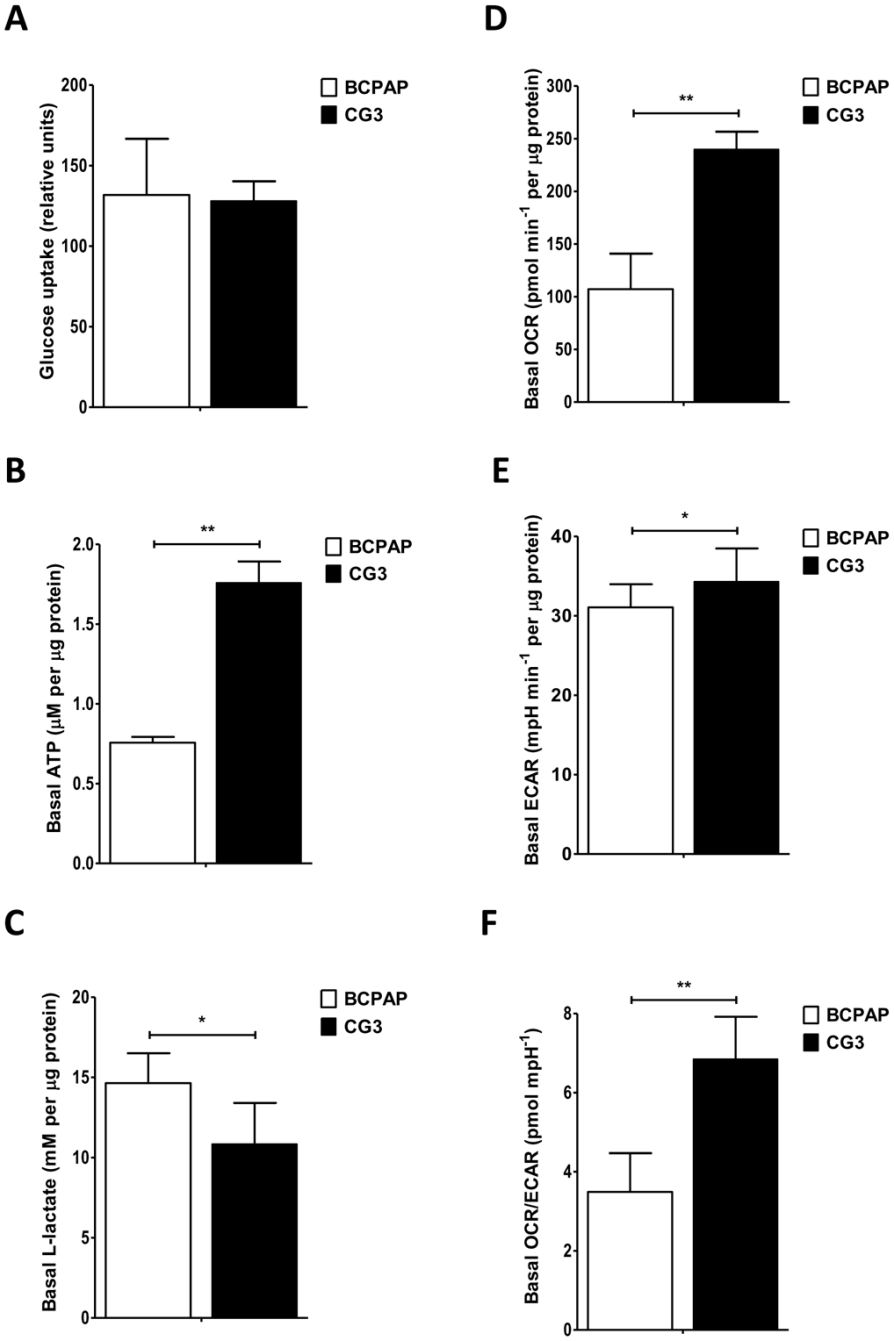
Bioenergetics in BCPAP and CG3 papillary thyroid carcinoma (PTC) cell lines. (A) Basal glucose uptake. (B) Basal intracellular ATP levels. (C) Basal extracellular l-lactate production. (D) Basal oxygen consumption rate (OCR). (E) Basal extracellular acidification rate (ECAR). (F) Basal OCR/ECAR ratio. Student’s *t* test was used to compare BCPAP and CG3 cells (*p < 0.05, **p < 0.001). Data are presented as means ± standard deviation (SD).

To identify potential differences in the metabolic and energy requirements of these two PTC cell lines, their basal glucose uptake, intracellular ATP production, extracellular l-lactate production, oxygen consumption rate (OCR), and extracellular acidification rate (ECAR) were first analyzed in real-time using the Seahorse X-24 flux analyzer. Although glucose uptake in BCPAP and CG3 cells was similar (Fig 1A), intracellular ATP levels were significantly lower (< 50%) (Fig 1B), and extracellular l-lactate production was significantly higher (Fig 1C) in BCPAP cells than in CG3 cells. We also measured basal cellular OCR, resulting from OXPHOS [35], and ECAR, which is related to glycolytic metabolism [35], in these two cell lines. Basal OCR, ECAR, and the OCR/ECAR ratio were significantly lower in BCPAP cells than in CG3 cells (Fig 1D–1F). For the basal OCR and the OCR/ECAR ratio, the values in BCPAP cells were less than half of those in CG3 cells (Fig 1D and 1F).

### Effect of 2-DG on cell viability in PTC cell lines

**Fig 2:**
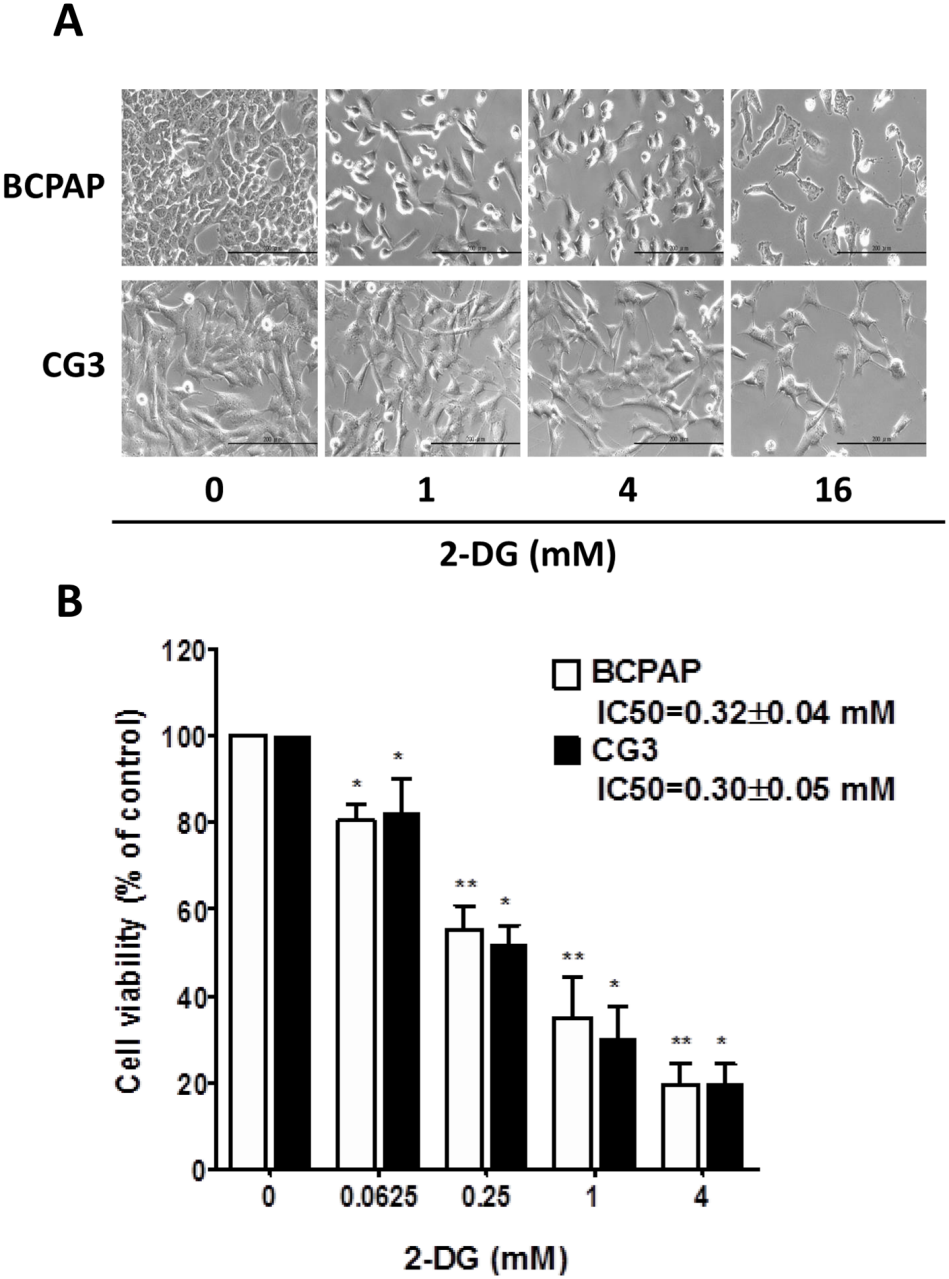
Susceptibility of PTC cells to 2-DG. (A) Morphological changes were observed using a microscopy (magnification: 200×). BCPAP and CG3 cells were treated for 48 h with 0, 1, 4, and 16 mM 2-deoxy-d-glucose (2-DG). (B) BCPAP and CG3 cells were treated for 48 h with 0, 0.0625, 0.25, 1, and 4 mM 2-DG. Cell viability was measured using a WST-1 assay. Data are shown as a percentage of the controls (untreated) cells. Student’s *t* test was used to compare the controls and treated cells. *p < 0.05, **p < 0.001, compared to the controls. Data are presented as means ± standard deviation (SD).

Given the differences in the bioenergetics of BCPAP and CG3 cells, we compared their responses to different doses of 2-DG, Phase-contrast images showed a decrease in cell number when both cell lines were treated for 48 h with 2-DG alone (1, 4, 16 mM) (Fig 2A). Dose-dependent reductions in cell viability were recorded in BCPAP and in CG3 cells (Fig 2B), without significant differences in the absolute half maximal inhibitory concentration (IC_50_) of 2-DG-treated cells: 0.32 ± 0.04 mM and 0.30 ± 0.05 mM, respectively (Fig 2B).

### Effects of 2-DG on the changes of bioenergetic functions of PTC cell lines

**Fig 3:**
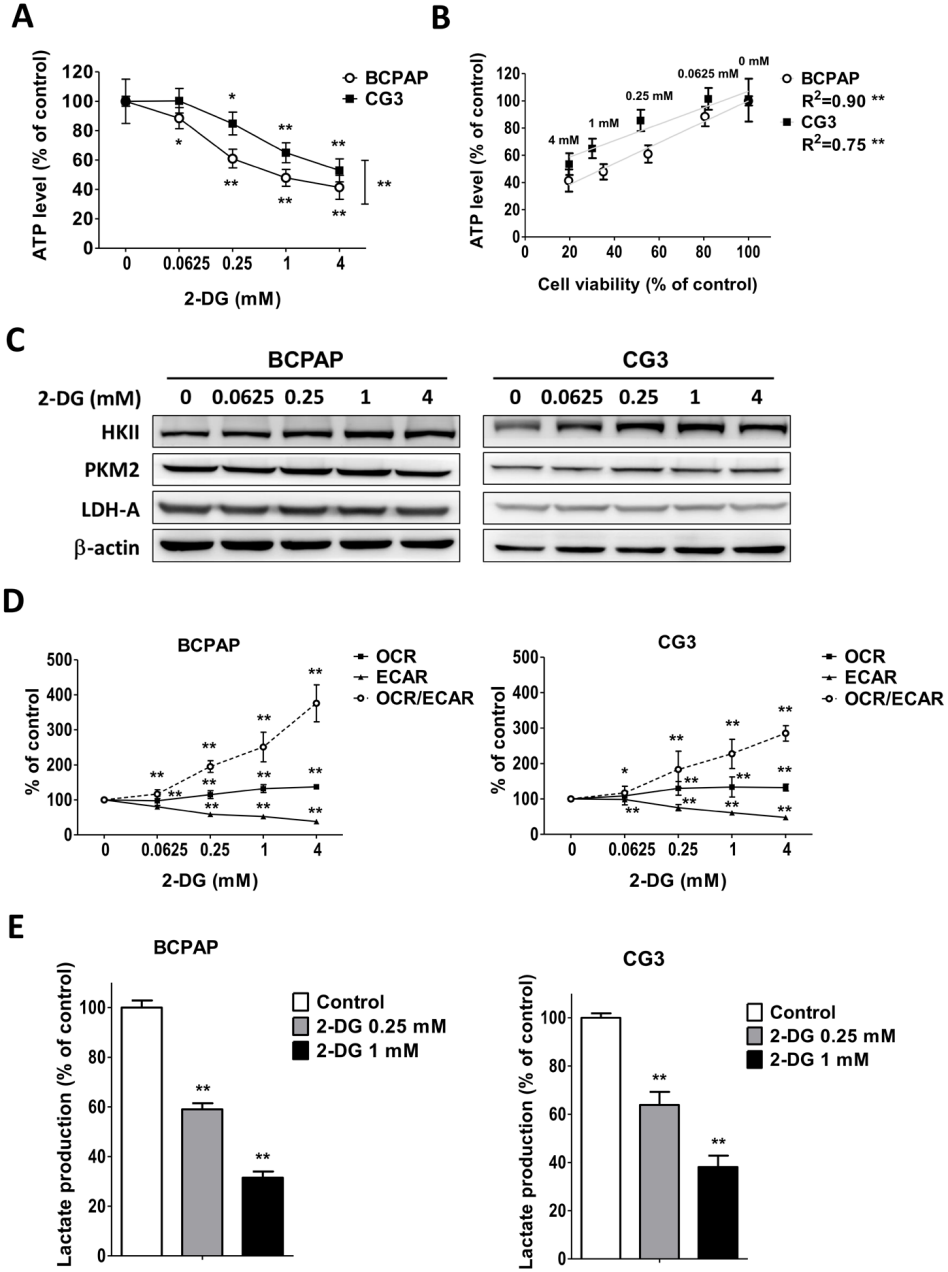
Effects of 2-DG on the changes in bioenergetics of PTC cells. (A) Intracellular ATP levels in cells treated for 48 h with different concentrations of 2-DG. Data are shown as a percentage of the controls (untreated) cells after normalization to total cellular protein in each well. *p < 0.05, **p < 0.001, compared to the controls (*t* test); ##*p* < 0.001, comparison of the two groups in a two-way analysis of variance (ANOVA). (B) Relationship between cell viability and intracellular ATP depletion. *p < 0.05, **p < 0.001, analyzed using linear regression. (C) Expression of hexokinase II (HKII), the M2 isoform of pyruvate kinase (PKM2), and lactate dehydrogenase-A (LDH-A) protein in BCPAP and CG3 cells treated for 48 h with 0, 0.0625, 0.25, 1, and 4 mM 2-DG. (D) OCR, ECAR, and OCR/ECAR in BCPAP and CG3 cells treated for 3 h with different concentrations of 2-DG. The changes were normalized to 1 μg cellular protein and are presented as a percentage of the control values. (E) Extracellular l-lactate production in BCPAP and CG3 cells treated for 48 h with 0, 0.25, and 1 mM 2-DG. C-E: *p < 0.05, **p < 0.001, compared to the controls (*t* test). Data are presented as means ± standard deviation (SD).

ATP depletion following 2-DG exposure was significantly greater in BCPAP cells than in CG3 cells (Fig 3A) (*p* < 0.001). The correlation between cell viability and intracellular ATP depletion was significant (*p* < 0.0001) in both 2-DG-treated BCPAP cells (R^2^ = 0.90) and 2-DG-treated CG3 cells (R^2^ = 0.75) (Fig 3B). To quantify the induction of glycolytic genes, the mRNA levels of HKII, ENO1, GAPDH, PKM2, and LDH-A in cells treated for 48 h with 0–4 mM 2-DG were measured using real-time RT-PCR (S1 Fig). HKII mRNA expression was significantly reduced in BCPAP cells treated with 0.025 mM 2-DG and in CG3 cells treated with 0.0625 mM and 0.25 mM 2-DG. LDH-A mRNA expression was significantly reduced in BCPAP cells treated with 4 mM 2-DG. Both GAPDH and PKM2 mRNA levels were mildly increased in BCPAP and in CG3 cells treated with 0.0625–4 mM and 0.25–4 mM 2-DG, respectively. However, in all cases, the differences in the mRNA expression levels between the controls and treated cells were < 1.5 fold. Western blotting showed no significant changes in the expression of HKII, PKM2, or LDH-A protein in BCPAP or CG3 cells 48 h after their exposure to 2-DG (Fig 3C). In 2-DG-treated BCPAP and CG3 cells, the ECAR decreased concomitantly with increases in the OCR and in the OCR/ECAR ratio. The responses were dose-dependent (Fig 3D). A significant, dose-dependent decrease in extracellular l-lactate production was observed in both cell lines (Fig 3E).

### Effects of 2-DG plus doxorubicin on cell viability and colony formation in PTC cell lines

**Fig 4:**
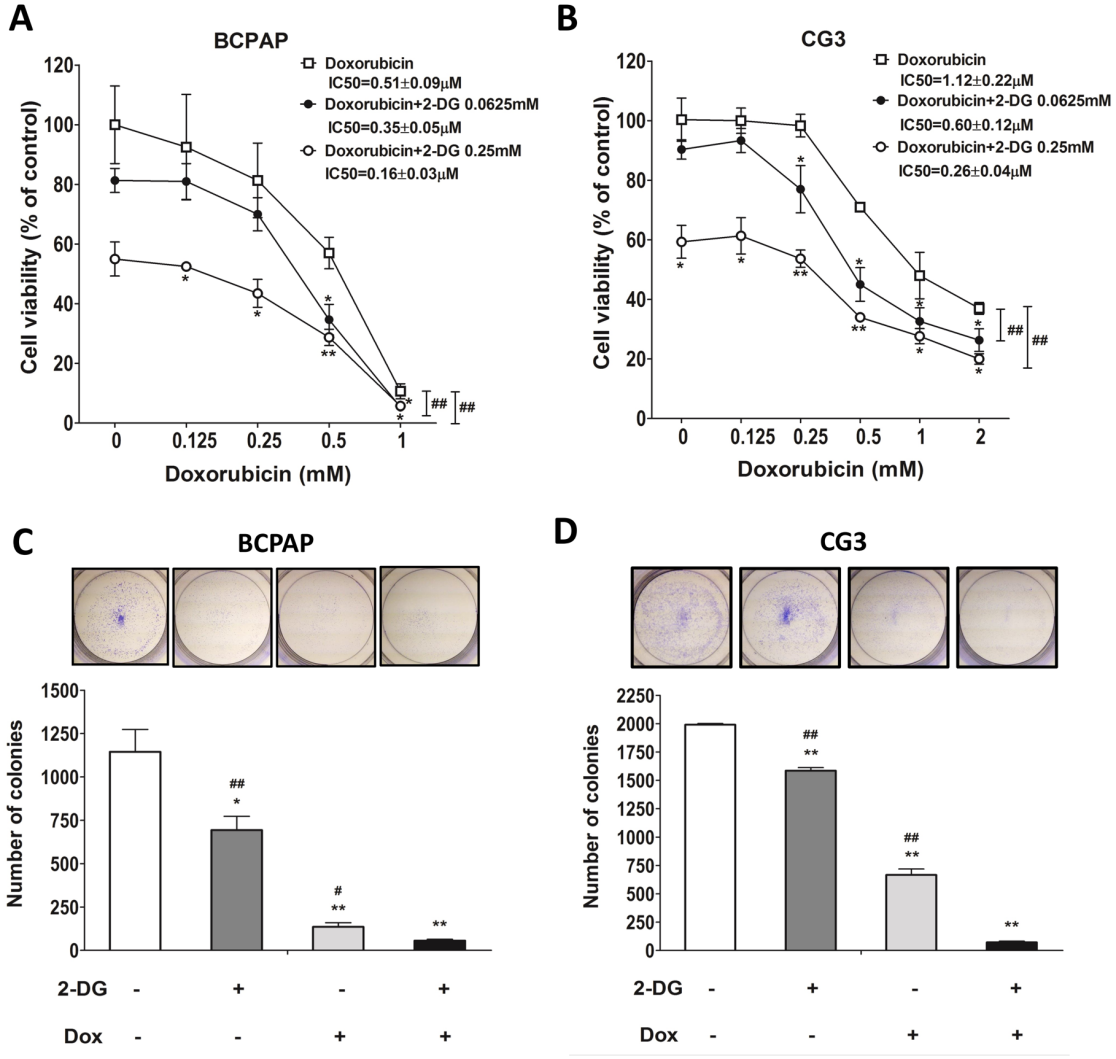
Effects of doxorubicin on the viability of PTC cells treated or not with 2-DG. (A, B) BCPAP and CG3 cells cultured in medium supplemented with 10% fetal bovine serum were treated for 48 h with different concentrations of doxorubicin in the presence or absence of 2-DG. Post-treatment, cell viability was measured using a WST-1 assay. (C, D) Colony formation in BCPAP and CG3 cells treated for 48 h with 1 mM 2-DG, 0.5 μM doxorubicin, or both. The medium was replaced with fresh medium for 3–5 days to check for colony formation. *p < 0.05, **p < 0.001, compared to the controls (*t* test); panels A, B: ##*p* < 0.001, 2-way ANOVA; C, D: ##*p* < 0.001, compared to 2-DG plus doxorubicin treatment (*t* test). Data are presented as means± standard deviation (SD).

The growth of the PTC cell lines treated with doxorubicin alone for 48 h was only mildly inhibited and in both cell viability was still > 60% at the concentration of 0.5 μM (Fig 4A and 4B). The IC_50_ of doxorubicin alone in CG3 cells was 2.2 times higher than that in BCPAP cells. To determine whether 2-DG increased the cytotoxic activity of doxorubicin, the cells were treated with 2-DG (0.0625 mM or 0.25 mM) plus doxorubicin at various concentrations. The IC_50_ of doxorubicin plus 2-DG was significantly lower in BCPAP cells and in CG3 cells treated with doxorubicin alone: 0.51 ± 0.09 μM and 1.12 ± 0.22 μM, respectively. The IC_50_ of doxorubicin plus 0.0625 mM 2-DG was 0.35 ± 0.05 μM (69% of the IC_50_ of doxorubicin alone) in BCPAP cells and 0.60 ± 0.12 μM (53%) in CG3 cells. The IC_50_ of doxorubicin plus 0.25 mM 2-DG was 0.16 ± 0.03 μM (31%) in BCPAP cells and 0.26 ± 0.04 μM (23%) in CG3 cells (Fig 4A and 4B). For both BCPAP and CG3 cells, colony formation was significantly lower in cells treated with doxorubicin plus 2-DG than in the controls or in cells treated with either 2-DG or doxorubicin alone (Fig 4C and 4D).

### Effects of 2-DG plus sorafenib on cell viability and colony formation in PTC cell lines

**Fig 5:**
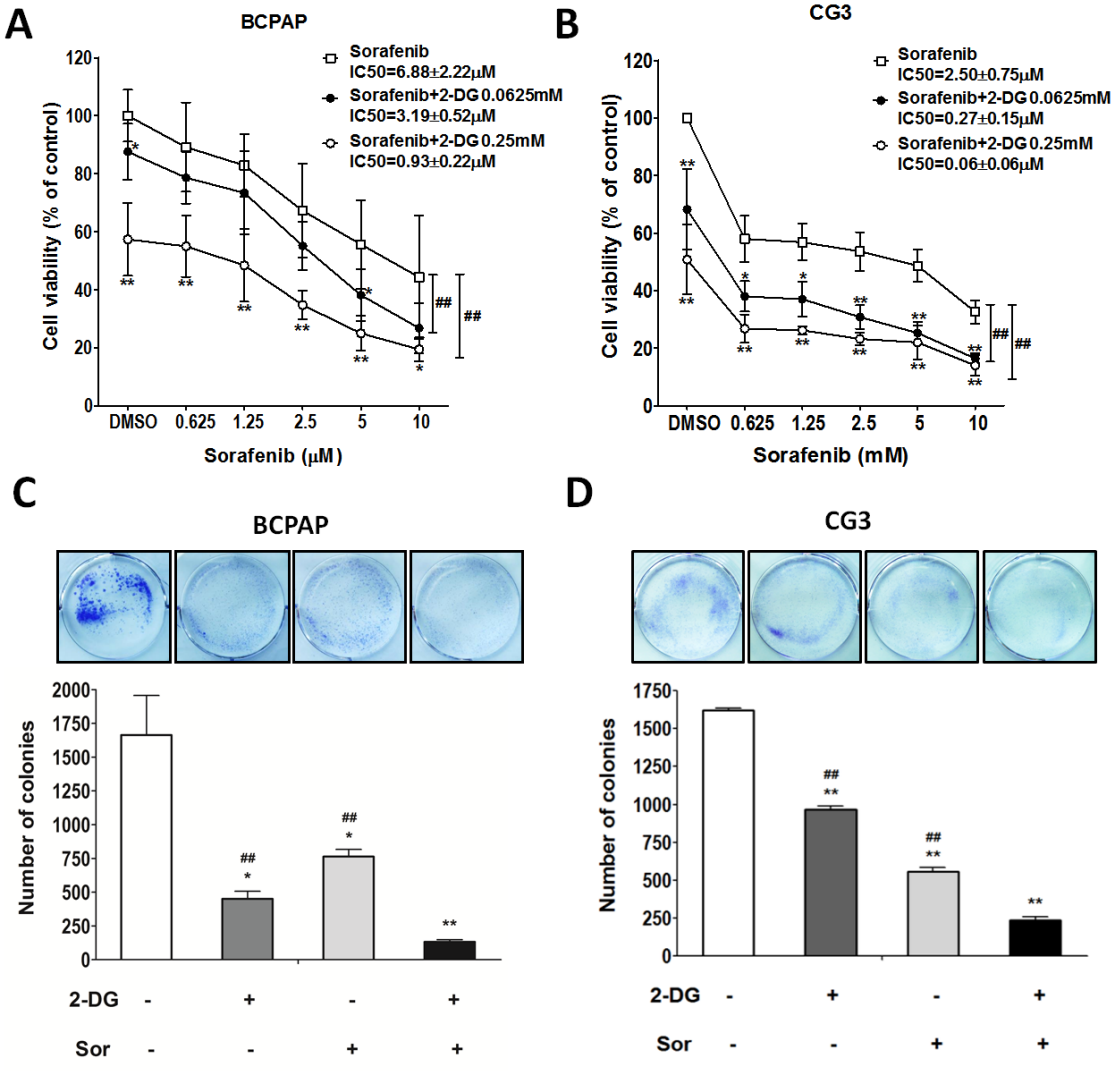
Effects of sorafenib on the viability of PTC cells treated or not with 2-DG. (A, B) BCPAP and CG3 cells cultured in medium supplemented with 10% fetal bovine serum were treated for 48 h with different concentrations of sorafenib in the presence or absence of 2-DG. Post-treatment, cell viability was measured using a WST-1 assay. (C, D) Colony formation in BCPAP and CG3 cells treated for 48 h with 0.25 mM 2-DG, 2.5 μM sorafenib, or both. The medium was replaced with fresh medium for 3–5 days to check for colony formation. *p < 0.05, **p < 0.001, compared to the controls (*t* test); A, B: ##*p* < 0.001, 2-way ANOVA; C, D: ##*p* < 0.001, compared to 2-DG plus sorafenib treatment (*t* test). Data are presented as means ± standard deviation (SD).

The growth of BCPAP cells treated with sorafenib alone for 48 h was only mildly inhibited, and the viability of both cell lines exposed to 2.5 μM sorafenib was still > 50% (Fig 5A and 5B). The IC_50_ of sorafenib alone was 2.7 times higher in BCPAP cells than in CG3 cells. BCPAP and CG3 cells were treated with 2-DG (0.0625 mM or 0.25 mM) plus sorafenib at various concentrations. The IC_50_ of sorafenib alone was 6.88 ± 2.22 μM in BCPAP cells and 2.50 ± 0.75 μM in CG3 cells. The IC_50_ of sorafenib plus 0.0625 mM 2-DG was 3.19 ± 0.52 μM (46% of the IC_50_ of sorafenib alone) in BCPAP cells and 0.27 ± 0.15 μM (10.8%) in CG3 cells. The IC_50_ of sorafenib plus 0.25 mM 2-DG was 0.93 ± 0.22 μM (13.5%) and 0.06 ± 0.06 μM (2.4%) in BCPAP and CG3 cells, respectively (Fig 5A and 5B). In both cell lines, colony formation was significantly lower in cells treated with sorafenib plus 2-DG than in controls and in cells treated with either 2-DG or sorafenib alone (Fig 5C and 5D).

### Effects of 2-DG plus doxorubicin or sorafenib on apoptosis and metabolic dysfunction in PTC cell lines

**Fig 6:**
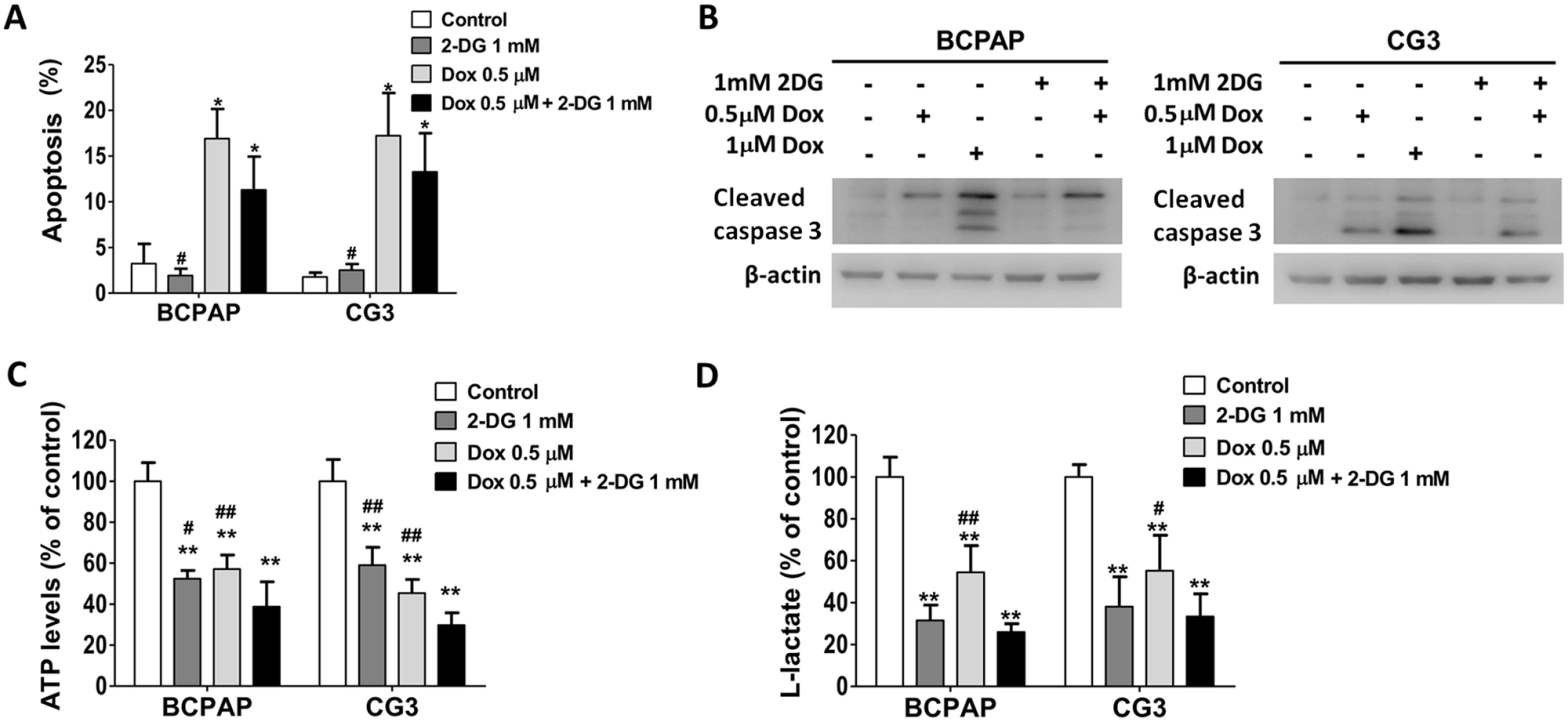
Effects of 2-DG, doxorubicin, or both on apoptosis and metabolic dysfunction in PTC cells. (A) The effect of 2-DG, doxorubicin, or both on apoptosis in BCPAP and CG3 cells. The two cell lines were cultured in medium supplemented with 10% fetal bovine serum and then treated for 48 h with 1 mM 2-DG, 0.5 μM doxorubicin, or both. Apoptosis was examined using propidium iodide (PI)/annexin V staining and flow cytometry; PI^−^/annexin V^+^ cells were defined as apoptotic cells. (B) The expression of cleaved caspase 3 in BCPAP and CG3 cells treated for 48 h with 2-DG, doxorubicin, or both. (C) Intracellular ATP levels and (D) extracellular l-lactate production in cells treated for 48 h with 1 mM 2-DG, 0.5 μM doxorubicin, or both. *p < 0.05, **p < 0.001, compared to the controls (*t* test); #*p* < 0.05, ##*p* < 0.001, compared to 2-DG plus doxorubicin treatment (*t* test). Data are presented as means ± standard deviation (SD).

BCPAP and CG3 cells were double-stained with annexin V and propidium iodide (PI) and then analyzed using flow cytometry to investigate apoptosis (defined as PI^−^/annexin V^+^). Western blotting was used to analyze the expression level of cleaved caspase-3. Cells treated with doxorubicin (0.5 μM) alone showed significantly higher levels of apoptosis and cleaved caspase-3 (Fig 6A and 6B). In neither cell line did 2-DG (1.0 mM) alone induce apoptosis or cleaved caspase-3 expression. Apoptosis and cleaved caspase-3 expression levels were not significantly different in cells treated for 48 h with 2-DG (1.0 mM) plus doxorubicin (0.5 μM) vs. doxorubicin (0.5 μM) alone (Fig 6A and 6B). Extracellular l-lactate and intracellular ATP levels were significantly lower in the two cell lines after treatment with 2-DG, doxorubicin, or both for 48 h (*p* < 0.001) (Fig 6C and 6D). Intracellular ATP levels were significantly lower in cells treated with 2-DG plus doxorubicin than in controls and in cells treated with 2-DG only or doxorubicin only (Fig 6C). Flow cytometry of cells treated with 2-DG, sorafenib, or both for 48 h and then double-stained with annexin V/PI showed no significant differences in apoptosis levels (data not shown).

## Discussion

A number of studies have reported that *BRAF* gene mutations, especially *BRAF* ^*V600E*^, are associated with the aggressiveness of PTC [36,37]. In our study, the BCPAP cell line, expressing the *BRAF* ^*V600E*^ mutation, because of its higher extracellular l-lactate production, lower intracellular ATP level, lower OCR level, and lower OCR/ECAR ratio, was more glycolytic than the CG3 cell line, which does not carry this mutation. Our findings are consistent with those of other studies [38,39] showing changes in the glucose metabolic pathway, including GLUT1 and PKM2 overexpression, in PTCs with *BRAF* mutations. These cells have a selective growth advantage through activation of aerobic glycolysis.

The antitumor activity of 2-DG has been demonstrated in various cancer cell lines and in murine cancer models [19–25]. Although in other studies [10,23] cell lines that were more glycolytic were also more sensitive to 2-DG, we found similar effects on cell viability in BCPAP and CG3 cells treated with 2-DG alone. Fluctuating HKII, ENO1 and LDH-A mRNA expression and the increased expression of GAPDH and PKM2 mRNA were detected in both BCPAP and CG3 cells treated for 48 h with 2-DG. The difference in gene expression between control and treated cells was consistently less than 1.5 fold. The results are similar to those obtained for the expression of HKII, PKM2, and LDH-A protein in BCPAP or CG3 cells treated for 48 h with 2-DG. The minor difference in mRNA vs. protein expression can be explained by the quality control of translation and mRNA stability determinants, such as poly (A) length, the 5’ cap structure of mRNA, trans-acting factors controlling mRNA turnover, and nonsense-mediated decay [40,41]. Intracellular ATP and extracellular l-lactate levels were significantly depleted in 2-DG-treated BCPAP cells and CG3 cells. The decreased ECAR and the increased OCR and OCR/ECAR ratio in both cell lines in response to 2-DG demonstrated the shift to a predominantly OXPHOS-dependent metabolism. Because 2-DG blocks the first step of the glycolytic pathway, this shift did not induce a simultaneous increase in intracellular ATP levels. The findings were more prominent in the more glycolytic BCPAP cells. A good correlation between cell viability and intracellular ATP depletion in both PTC cell lines demonstrated that cell proliferation critically depends on ATP generation.

The IC_50_ study of doxorubicin and sorafenib showed the different sensitivities of BCPAP and CG3 cells to these two anticancer agents, as the former were more sensitive to doxorubicin and the latter were more sensitive to sorafenib. Treatment of both cell lines with 2-DG plus doxorubicin significantly reduced the IC_50_ of doxorubicin, even with low doses of 2-DG (0.0625 mM and 0.25 mM). 2-DG as an adjuvant agent has been studied in breast and anaplastic thyroid cancers [42,43]. Our results support the findings of those studies, that 2-DG plus doxorubicin is a potentially effective adjunct therapy for PTC. The advantage of 2-DG in lowering the required dose of chemotherapeutic agents and the cytotoxicity of radiotherapy is that cancer-free cells are protected against damage, including doxorubicin-induced cardiac damage [44,45].

The most common side effects of sorafenib are skin toxicity (predominantly hand-foot skin reactions, a variety of rashes, and alopecia), gastrointestinal disturbances, constitutional adverse reactions, and hypertension. Although most adverse reactions are manageable, > 50% of patients require a dose reduction [18]. In this study, when sorafenib was combined with 0.0625 mM 2-DG, its IC_50_ decreased to 46% in BCPAP cells and to 10.8% in CG3 cells; when combined with 0.25 mM 2-DG, its IC_50_ decreased dramatically, to 13.5% in BCPAP cells and to 2.4% in CG3 cells. These findings suggest a novel approach to the treatment of PTC by combining 2-DG and lower doses of sorafenib. However, the results of our *in vitro* studies required further validation by animal studies before they can be tested in humans.

Monotherapy with 2-DG alone was unable to cause apoptosis in either of the PTC cell lines; rather, it inhibited cell proliferation by causing cell cycle arrest at G0/G1 (data not shown). The combination of 2-DG with doxorubicin or with sorafenib in BCPAP and CG3 cells treated with these drugs for 48 h showed no synergistic or antagonistic effects on apoptosis or the expression of cleaved caspase-3, despite a significant depletion of intracellular ATP. Additional studies, including the use of microarray and proteomics, are necessary to clarify the underlying mechanisms.

Taken together, our findings indicate that BCPAP cells are more glycolytic than CG3 cells and that energy depletion is important for inhibiting PTC cell proliferation. 2-DG increased chemosensitivity to doxorubicin and to sorafenib in both cell lines. Therefore, low doses of 2-DG and either doxorubicin or sorafenib, may provide anticancer effects otherwise achievable only with high doses of the latter chemotherapeutic agents, regardless of the *BRAF* ^*V600E*^ mutation. One of the advantages of 2-DG in lowering the required dose of doxorubicin or sorafenib is the protection of non-cancer cells. This conclusion remained to be validated by future work, including additional experiments using primary PTC cell cultures from patients or other PTC cell lines to anticipate individual differences in the response to this therapeutic approach.

## Supporting Information

S1 Fig Effect of 2-DG on the mRNA expression of glycolytic genes in PTC cells. Relative expression of hexokinase II (HKII), glyceraldehyde-3-phosphate dehydrogenase (GAPDH), enolase 1 (ENO1), M2 isoform of pyruvate kinase (PKM2), and lactate dehydrogenase-A (LDH-A) mRNA in BCPAP and CG3 cells treated for 48 h with 0, 0.0625, 0.25, 1, and 4 mM 2-DG. *p < 0.05, compared to controls (*t* test). The data are presented as means ± standard deviation (SD).
(TIF)

S1 File Supplementary Methods. RNA isolation, RT-PCR, qRT-PCR, and primers.
(DOCX)
