# Supplementary figures and images for: 2-Deoxy-d-Glucose Can Complement Doxorubicin and Sorafenib to Suppress the Growth of Papillary Thyroid Carcinoma Cells
Source: PLoS One. 2015 Jul 2;10(7):e0130959. doi: 10.1371/journal.pone.0130959 (PMC4489888; doi:10.1371/journal.pone.0130959)

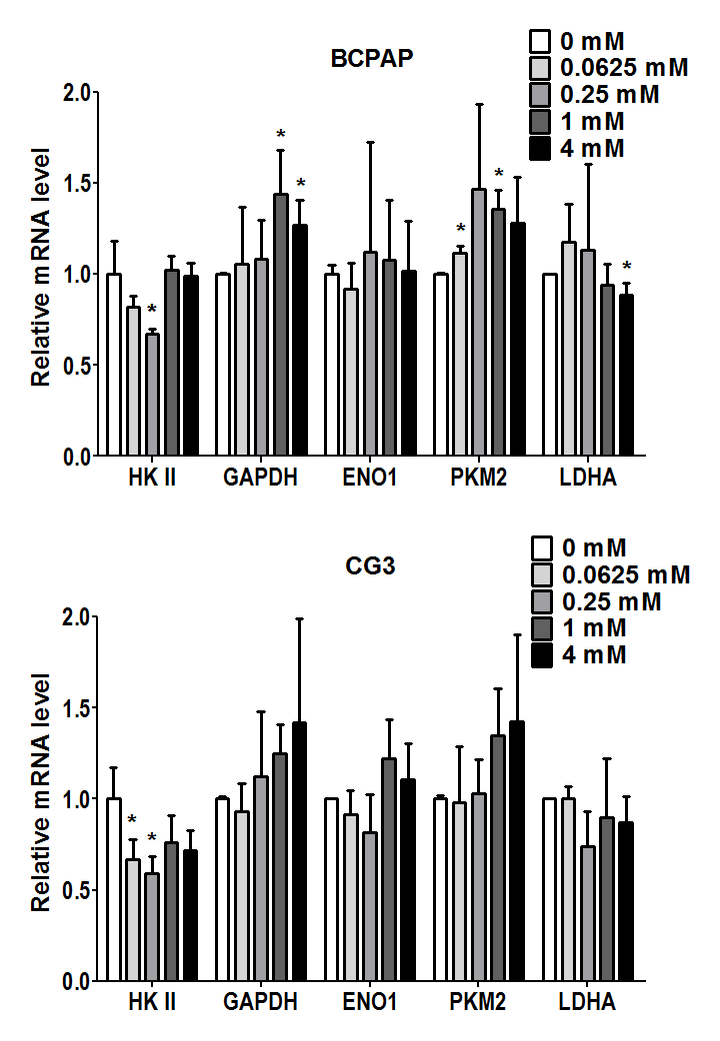

Supplement: S1 Fig — Relative expression of hexokinase II (HKII), glyceraldehyde-3-phosphate dehydrogenase (GAPDH), enolase 1 (ENO1), M2 isoform of pyruvate kinase (PKM2), and lactate dehydrogenase-A (LDH-A) mRNA in BCPAP and CG3 cells treated for 48 h with 0, 0.0625, 0.25, 1, and 4 mM 2-DG. *p < 0.05, compared to controls (t test). The data are presented as means ± standard deviation (SD). (TIF) [file pone.0130959.s001.tif]
