# Supplementary material for: 2-Deoxy-d-Glucose Can Complement Doxorubicin and Sorafenib to Suppress the Growth of Papillary Thyroid Carcinoma Cells
Source: PLoS One. 2015 Jul 2;10(7):e0130959. doi: 10.1371/journal.pone.0130959 (PMC4489888; doi:10.1371/journal.pone.0130959)
Supplement: S1 File — RNA isolation, RT-PCR, qRT-PCR, and primers. (DOCX) [file pone.0130959.s002.docx]

**Supporting Information**

**Supplementary Methods**

**RNA isolation, RT-PCR, qRT-PCR, and primers**

BCPAP and CG3 cells were treated for 48 h with 0–4 mM 2-DG. Total RNA was extracted from the cells with TRIzol reagent (Invitrogen) following the manufacturer’s instructions, and RNA was reverse transcribed into cDNA with random primers. The expression levels of selected genes were determined using the StepOnePlus™ real-Time PCR System (Applied Biosystems). β-actin was served as an internal control. The primers used for real-time PCR were:

β-actin-F 5’-AGCGCGGCTACAGCTTCA-3’;

β-actin-R 5’-GGCCATCTCTTGCTCGAAG-3’;

HKII-F 5’-TGGAGATGGAGAATCAGA-3’;

HKII-R 5’-CCAGGAAACTCTCGTCTA-3’;

GAPDH-F 5’-GGGCATCCTGGGCTACACT-3’;

GAPDH-R 5’-GGTGGTCCAGGGGTCTTACTC-3’;

ENO1-F 5’-AATGATAAGACTCGCTATATG-3’;

ENO1-R 5’-CGTTCAGTTTCTTGCTAA-3’;

PKM2-F 5’-TCGGAGGTTTGATGAAAT-3’;

PKM2-R 5’-TCTCCAGCATCTGAGTAG-3’;

LDHA-F 5’-GGTTGAGAGTGCTTATGA-3’;

LDHA-R 5’-AACACTAAGGAAGACATCA-3’.

The real-time PCR for each sample was performed three times. Independent experiments were done in triplicate.
